# Clustering of Pan- and Core-genome of *Lactobacillus* provides Novel Evolutionary Insights for Differentiation

**DOI:** 10.1186/s12864-018-4601-5

**Published:** 2018-04-24

**Authors:** Raffael C. Inglin, Leo Meile, Marc J. A. Stevens

**Affiliations:** 1 Laboratory of Food Biotechnology, Institute of Food, Nutrition and Health, ETH Zurich, Schmelzbergstrasse 7, 8092 Zurich, Switzerland; 2 Present address: Institute for Food Hygiene and Safety, University of Zurich, Winterthurerstrasse 272, 8057 Zurich, Switzerland

**Keywords:** Comparative genomics, *Lactobacillus*, Core-genome, Pan-genome, Horizontal gene transfer

## Abstract

**Background:**

Bacterial taxonomy aims to classify bacteria based on true evolutionary events and relies on a polyphasic approach that includes phenotypic, genotypic and chemotaxonomic analyses. Until now, complete genomes are largely ignored in taxonomy. The genus *Lactobacillus* consists of 173 species and many genomes are available to study taxonomy and evolutionary events.

**Results:**

We analyzed and clustered 98 completely sequenced genomes of the genus *Lactobacillus* and 234 draft genomes of 5 different *Lactobacillus* species, i.e. *L. reuteri*, *L. delbrueckii*, *L. plantarum*, *L. rhamnosus* and *L. helveticus.* The core-genome of the genus *Lactobacillus* contains 266 genes and the pan-genome 20′800 genes. Clustering of the *Lactobacillus* pan- and core-genome resulted in two highly similar trees. This shows that evolutionary history is traceable in the core-genome and that clustering of the core-genome is sufficient to explore relationships. Clustering of core- and pan-genomes at species’ level resulted in similar trees as well. Detailed analyses of the core-genomes showed that the functional class “genetic information processing” is conserved in the core-genome but that “signaling and cellular processes” is not. The latter class encodes functions that are involved in environmental interactions. Evolution of lactobacilli seems therefore directed by the environment. The type species *L. delbrueckii* was analyzed in detail and its pan-genome based tree contained two major clades whose members contained different genes yet identical functions. In addition, evidence for horizontal gene transfer between strains of *L. delbrueckii, L. plantarum,* and *L. rhamnosus,* and between species of the genus *Lactobacillus* is presented*.* Our data provide evidence for evolution of some lactobacilli according to a parapatric-like model for species differentiation.

**Conclusions:**

Core-genome trees are useful to detect evolutionary relationships in lactobacilli and might be useful in taxonomic analyses. *Lactobacillus*’ evolution is directed by the environment and HGT.

**Electronic supplementary material:**

The online version of this article (10.1186/s12864-018-4601-5) contains supplementary material, which is available to authorized users.

## Background

In the last 10 years, sequencing of complete genomes developed from research that required a consortium to an effort that a single person can manage [1]. The decreasing costs and increasing speed of complete genomes sequencing have led to an enormous amount of data of various quality that is not completely analyzed yet [2]. Next generation sequencing (NGS) technology is highly useful for research on diseases or on phenotypic variations of specific genes. Such research; however, needs high quality data, i.e. a genome coverage that results in a reliable sequence [3]. In microbiology, high quality sequences are suitable for research such as sub-typing of *Salmonella enterica* strains to monitor outbreaks or for calculating bacterial pan-genomes [4, 5]. Complete genome sequences are only poorly applied in bacterial classification and phylogenetic studies [6]. Complete genomes are; however, most preferable for phylogenetic studies because evolutionary pressure works on the whole organism and not on a subset of genes.

A polyphasic approach is commonly used for bacterial classification and analysis of evolutionary relationships [7, 8]. Polyphasic approaches are not standardized and include phenotypic, genotypic and chemotaxonomic parameters to determine whether a bacterial isolate belongs to an existing species or if a new species has to be defined [6, 9]. Assignment of a strain to a species is based amongst others on two genotypic parameters: sequence similarity of more than 98.7% in the 16S rRNA gene and a DNA-DNA hybridization (DDH) degree of more than 70% [10, 11]. Other genetic parameters are also useful for bacterial classification. For example, EcoSNPs, SNPs that are specific for a dimorphic nucleotide position in a clade, can be used to build phylogenetic trees [12]. Comparison of complete genome sequences to 70% DNA-DNA hybridization levels is possible using defined parameters for conserved DNA regions and unique matches [13–15]. Additionally, an average nucleotide identity (ANI) value of 94% corresponds to 70% DNA-DNA hybridization and is thus also a usable parameter to define species.

The core-genome is the set of homologous genes that are present in all genomes of an analyzed dataset and the pan-genome is the set of all genes that are present in the analyzed dataset [16]. In addition, the softcore-genome is the set of genes, present in ≥95% of the genomes [17]. The softcore-genome is useful, because it circumvents the absolute impact of poor quality sequences on the core-genome. An open pan-genome is increasing with every new genome included whereas a closed pan-genome remains on a constant gene number after a certain number of genomes were included [18]. The status of the core- and pan-genomes depends on number of analyzed genomes and on the properties of the species analyzed, such as the ability of the species to integrate exogenous DNA and on the species’ lifestyle and environment [19–21]. Taxonomy of bacteria based on core- and pan-genome might be a powerful extension of the polyphasic approach. Pan-genome clustering of 29 *Geobacillus* genomes revealed horizontal gene transfer as a factor in evolution of *Geobacillus* and such transfer should be implemented in its taxonomy [22]. Horizontal gene transfer was also detected in a recently diverged *Vibrio* population, where ecological differentiation based on single nucleotide polymorphisms occurred [12].

The heterogeneous genus *Lactobacillus (L.)* contains 173 species not including 17 subspecies [23]. Lactobacilli have been isolated from a whole range of fermented food products such as yoghurt, cheese, vegetables, beverages, sausages and sourdough. Further, lactobacilli are also found in the human and animal gastro-intestinal tracts [24]. The Qualified Presumption of Safety (QPS) status from the European Food Safety Agency EFSA facilitates commercial use and acceptance of most *Lactobacillus* species. This makes them ideal candidates for the use as protective and starter cultures [25]. Aside from their preserving qualities, strains of some *Lactobacillus* species are also exploited for their health promoting potential as probiotics and vaccine carrier [26, 27]. In December 2016, a total of 121 completely sequenced and assembled genomes were available in public databases with sizes ranging from 1.37 Mpb for *L. sanfranciscensis* TMW1.1 to 3.74 Mbp for *L. paracellinoides* TMW1.1995 [28]. *Lactobacillus* and related genera were initially clustered into three subgroups based on 16S RNA gene comparison: the *Lactobacillus delbrueckii* group, the *Lactobacillus casei-Pediococcus* group and the *Leuconostoc* group [29, 30]. A recent 16S rRNA gene based clustering of the *Lactobacillus* type strains species resulted in a phylogenetic tree with 15 major groups [31]. There is; however, only moderate correlation between 16S rRNA gene sequence clustering and clustering based on fermentation type and metabolic properties.

The goal of this study was to analyze the phylogeny of the *Lactobacillus* genus and a dedicated set of species via core-, softcore- and pan-genome clustering. Such complete genome based clustering provides a detailed overview of gene contents of the core- and pan-genome and will provide insights on relationship of species and their gene exchange.

## Methods

### Genome sequences

A total of 98 complete sequenced *Lactobacillus* genomes and 202 draft genomes belonging to the species *Lactobacillus plantarum*, *Lactobacillus helveticus*, *Lactobacillus delbrueckii*, *Lactobacillus reuteri* and *Lactobacillus rhamnosus* were obtained from public databases (Additional file 1). To prevent too high impact of poorly assembled genomes for the *Lactobacillus* species calculation, draft genomes were only used if they fell within a range of ± 2σ around the average gene and protein number of the species.

### Calculation of core- and pan-genome

Orthologous cluster were created using the Perl-script collection GET_HOMOLOGOUS [32] applying the following for identification and clustering CDS into orthologous groups: *-E <* 1e-05 for blastp searches and *-C* 75% minimum alignment coverage. The core-genome was determined using the Ortho Markov Cluster algorithm (OMCL) [33] and the pan-genome using the OMCL algorithm with *–t* 0; reporting all clusters in the pan-genome. A pan-genome matrix was created using the script compare_clusters with the settings: *-d* including only OMCL data, *−m* produce intersection in pan-genome matrix.

The core-genome was defined by genes present in all genomes, the softcore by genes present in 95–100% of the genomes, the shell by genes present in more than 2 genomes but less than 95% of the genomes and the cloud genes present in 2 or less of the genomes and calculated with the parse_pangenome_matrix script: *-s* report clusters.

The development-calculation of core- and pan-genome starts with comparing two genomes and including single genomes step-by-step until all genomes are integrated. The order of the included genome was randomized n-times (n = number of included genomes) and calculated with a home-made script in MATLAB R2014b based on the pangenome_matrix_t0. The home-made scripts used in this study are available in Additional file 2.

### Clustering and analyses of core- and pan-genome

Protein-based clustering was performed with GET_HOMOLOGOUS [32] using the OMCL algorithm as follows: *-t* 0, *−t* all or *-t* n (*n* = 0.95 x number of included genomes) for clustering the pan-, core- and softcore-genome, *−M;* with the OMCL algorithm and – A; to create an average identity matrix. The created average identity matrix of clustered sequences was visualized using the script hcluster_matrix with the option *–d* gower; for selecting the gower distance calculation for clustering [34]. Core- and pan-genome (Additional file 1) were analyzed with the metagenome analysis tool GhostKOALA against “genus_prokaryotes + family_eukaryotes” database using the Brite, Pathway and Module reconstruction algorithm [35]. Brite reconstruction uses KEGG Brite hierarchies with combined sets of K numbers. Pathway reconstruction aligns gene to the KEGG pathway map and Module reconstruction uses sets of K numbers to evaluate if a block (pathway or structural complex) is complete. The relative increase of genes in a category compared to the complete increase of genes in core- and pan-genomes were calculated and analyzed with Fisher’s exact test in MATLAB R2014b (Additional file 2).

### Identification of clade specific genes

Identification of clade specific genes in a set of bacterial isolates was performed using the parse_pangenome_matrix script of GET_HOMOLOGOUS [32] with option; −*A* a list of genomes in one clade; option *–B* a list genomes of another clade to compare against; −g finding genes present in genomes of clade A and absent in genomes of clade B; −e find gene family expansions in A with respect to B. To determine if a gene encodes a unique function in a clade that is not compensated by isoenzymes in the other clade, the core-genome of the clade was compared with the pan-genome of all other clades using GhostKOALA [35]. The presence of isoenzymes was analyzed for each gene manually.

### Identification of representative genes for clustering the type species *Lactobacillus delbrueckii*

To identify which gene or set of genes represents most closely the pan-genome-phylogenetic tree of *L. delbrueckii,* the tree of each core gene was compared to the tree of the pan genome of *L. delbrueckii* using TOPD/FMTS [36] and CLC Workbench 8 (CLC Genomic, Aarhus, Denmark). Each homologous gene set from the core genome was imported as multi-entry FASTA into CLC Genomic Workbench 8. The genes were aligned using the “Create alignment” tool using standard parameters. Trees were created with the toolbox “Create Tree” using “Neighbor Joining” as tree construction method and “Jukes-Cantor” as nucleotide distance measure with a Bootstrap value of 100. Trees were exported as nexus files and compared to the pan-genome tree using TOPD/FMTS using the following parameters: *-m* nodal method of calculation; *−n* 10 number of random sequences; *−c* reference comparing all versus pan-genome tree. Identical trees have a nodal distance = 0. The higher the nodal distance is the less identical are the trees. The 5% and 95% percentile was calculated for all core gene nodal distances and the genes outside this range analyzed manually.

### Identification of ecoSNPs in *Lactobacillus delbrueckii*

To analyze ecoSNP distribution, core gene alignments were exported from the CLC workbench as ClustalW files and imported into MATLAB R2014b to determine the consensus sequences. Each gene was compared with the consensus sequence and SNPs were determined and analyzed for its specificity to a clade in the pan-genome of *L. delbrueckii*.

### Potential horizontal gene transfer within clades

For identification of potential horizontal gene transfer (HGT) events, genes with a 30–70% presence in all clades were selected. An absence-presence matrix for all genes and strains was constructed for each clade in MS Excel and genes within the 30–70% criterion selected.

## Results

### Calculation of core- and pan-genome of complete *Lactobacillus* genomes

**Fig. 1:**
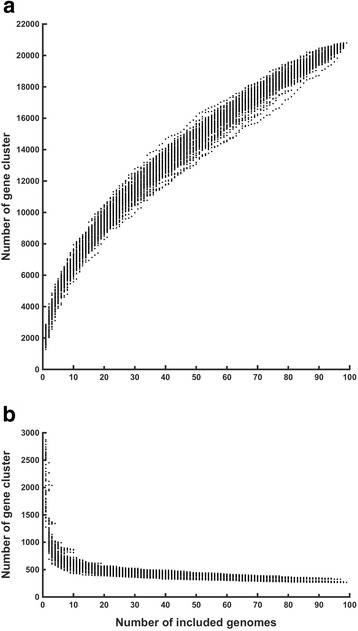
Pan- and core-genome evolution of *Lactobacillus.* **a** For every included genome the size of the pan-genome increases. **b** Evolution of core-genome of 98 complete *Lactobacillus* genomes. After 70 genomes, the size of the core-genome is only decreasing by a few genes per included genome. Order of calculation was randomized for 98 sets, each represented by a single point

**Fig. 2:**
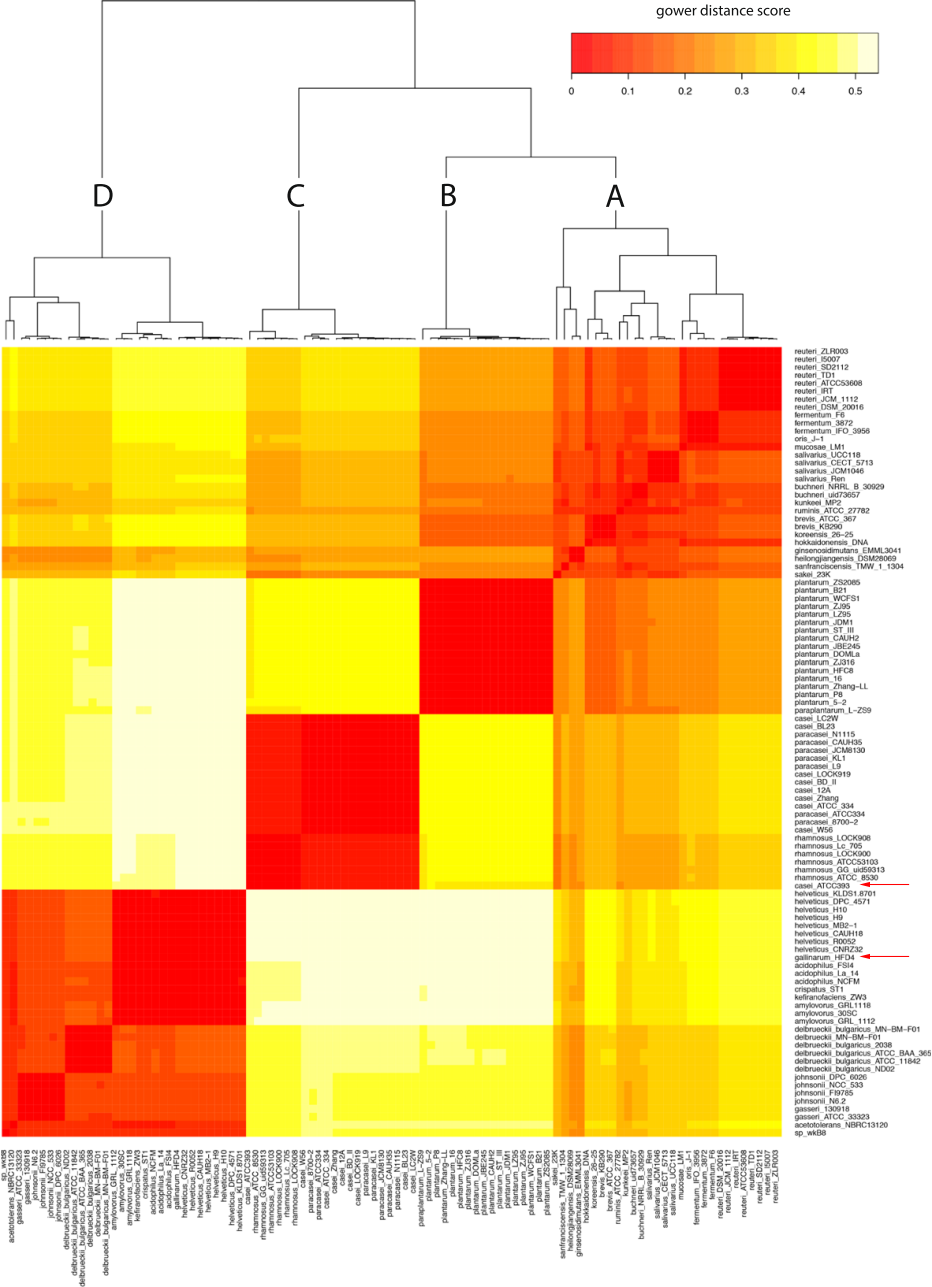
Core-genome clustering of genus *Lactobacillus.* Heatmap clustering according to 266 core genes from 98 *Lactobacillus* genomes. Gower distance score based on ANI: Red = more similar, white = less similar, Outliers are marked with red arrows. **a** reuteri-fermentum-salivarius clade, (**b**) plantarum clade, (**c**) casei-paracasei-rhamnosus clade and (**d**) helveticus-delbrueckii-johnsonii clade

**Fig. 3:**
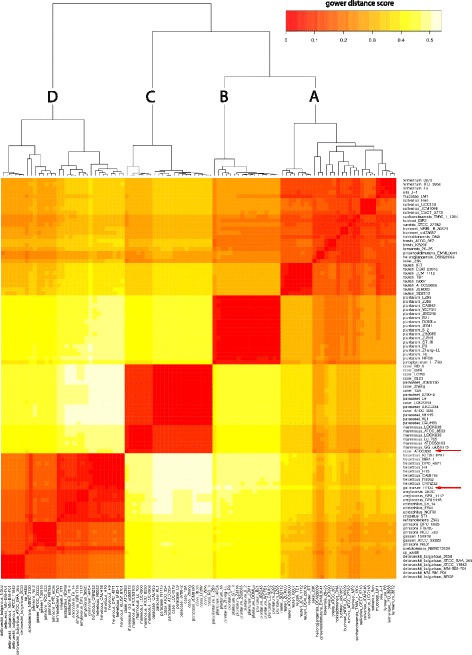
Pan- genome clustering of genus *Lactobacillus.* Heatmap clustering according to 20′800 pan-genome genes from 98 *Lactobacillus* genomes. Gower distance score based on ANI: Red = more similar, white = less similar, Outliers are marked with red arrows. **a** reuteri-fermentum-salivarius clade, (**b**) plantarum clade, (**c**) casei-paracasei-rhamnosus clade and (**d**) helveticus-delbrueckii-johnsonii clade

To obtain a general view of *Lactobacillus* genome contents, the core- and pan-genome for 98 completely assembled *Lactobacillus* genomes were calculated. The pan-genome for the *Lactobacillus* genus still increased with approximately 50 genes after addition of a 98th genome and thus can be considered as open (Fig. 1a). The core-genome rapidly decreased with the first set of genomes, but stabilizes after the 70^th^ is added, showing it’s closed (Fig. 1b). The core-genome contained 266 genes and the pan-genome 20′800 genes (Table 1; Additional files 3 and 4). A core-genome based clustering revealed 4 major clades: (A), a *reuteri-fermentum-salivarius* clade, (B), a *plantarum-paraplantarum* clade, (C) a *casei-paracasei-rhamnosus* clade and (D) a *helveticus-delbrueckii-johnsonii* clade (Fig. 2). The softcore- and pan-genome were also clustered and the 4 clades appeared again as separate clusters and contained the same isolates (Fig. 3). The highly similar pan- and core-genome clusters shows that evolutionary relationship appears already in the core genome. In general, species clustered together. However, some strains from the species *L. casei* / *L. paracasei* and *L. helveticus* / *L. gallinarum* did not.

**Table 1:** Core- and pan-genome of the genus *Lactobacillus* and of 5 *Lactobacillus* spec

Genus	Species	N _genomes_	Genome size (Mbp)	N_Genes_	core	softcore	shell	cloud	pan
*Lactobacillus*	*–*	98	2.47 ± 0.55	2274 ± 528	266	594	7249	12,957	20,800
*Lactobacillus*	*helveticus*	19	2.02 ± 0.13	2050 ± 164	908	1062	1133	1155	3350
*Lactobacillus*	*reuteri*	25	2.10 ± 0.12	2050 ± 117	897	1306	1364	1290	3960
*Lactobacillus*	*rhamnosus*	51	2.97 ± 0.08	2788 ± 71	811	1920	1736	1233	4889
*Lactobacillus*	*plantarum*	122	3.27 ± 0.13	3075 ± 140	1037	2144	2826	2640	7610
*Lactobacillus*	*delbrueckii*	29	1.88 ± 0.13	1873 ± 93	756	1042	1336	1082	3460

### Detailed analysis of strains with unexpected clustering

The *L. casei* type strain ATCC 393 did not clusters with other *L. casei* strains, but with 6 *L. rhamnosus* strains (Figs. 2 and 3). The genome of strain ATCC 393 contains 213 KEGG orthology (KO) assignments that are not present in any other of the 21 *L. casei*, *L. paracasei* and *L. rhamnosus* genomes. 11 of these 213 KOs are related to carbohydrate metabolism, 7 to environmental information processing and all other to hypothetical functions (Table 2). From 27 annotated KOs, 22 describe functions that are present in other *L. casei*, *L. paracasei* and *L. rhamnosus* isolates but are encoded by isogenes. However, *L. casei* ATCC 393 contains 5 KOs with a unique function, including one catalase (Table 3).

**Table 2:** Unique genes in *L. casei* ATCC393, *L. gallinarum* HDF4 and in *L. delbrueckii* strains as identified with GhostKOALA functional categories

KEGG orthology	*L. casei* ATCC393	*L. gallinarum* HDF4	*L. delbruecki* bulgaricus clade	*L. delbrueckii* diverse clade
Carbohydrate metabolism	11	5	7	1
Energy metabolism	0	0	2	1
Lipid metabolism	0	2	3	0
Nucleotide metabolism	1	0	0	1
Amino acid metabolism	1	3	3	3
Metabolism of other amino acid	1	0	0	0
Glycan biosynthesis and metabolism	2	0	0	0
Metabolism of cofactors and vitamins	0	3	0	1
Metabolism of terpenoids and polyketides	0	1	0	0
Biosynthesis of other secondary metabolites	0	1	0	0
Xenobiotics biodegradation and metabolism	0	0	3	0
Enzyme families	2	1	0	0
Genetic Information Processing	2	11	1	1
Environmental Information Processing	7	9	6	0
Cellular Processes	5	6	3	0
Organismal Systems	1	0	0	0
Human Diseases	1	2	2	0
Unclassified	5	9	0	1
Annotated KEGG orthologous	27^a^	38^a^	12^a^	5
Hypothetical function	186	143	30	5
Query dataset	213	181	42	10

^a^KEGG orthologous can be present in single or multiple categories

**Table 3:** Unique genes of *Lactobacillus* strains from Table 2 with no isoenzymes in the pan-genome that they were compared to, which would comply the same function. K-number according to KEGG database

Present in isolate	K-number	EC-number	Function
*L. casei* ATCC393	K01788	5.1.3.9	N-acylglucosamine-6-phosphate 2-epimerase
	K03781	1.11.1.6	Catalase
	K00681	2.3.2.2	gamma-glutamyltranspeptidase
	K00681	3.4.19.13	glutathione hydrolase
	K20997		polysaccharide biosynthesis protein (pslA)
*L. gallinarium* HFD4	K00278	1.4.3.16	L-aspartate oxidase (nadB)
	K00558	2.1.1.37	DNA (cytosine-5)-methyltransferase 1
	K03517	2.5.1.72	quinolinate synthase (nadA)
	K18231		Macrolide transporter symstem ATP-binding/permease protein (msrA)
*L. delbrueckii* diverse cluster	K00135	1.2.1.16	Succinate semialdehyde dehydrogenase
	K00926	2.7.2.2	carbamate kinase
	K00611	2.1.3.3	ortnithine carbamoyltransferase
	K02970		small subunit ribosomal protein S21

*L. zeae* was not included in the pan/core-genome analyses because a closed genome is not available for the species. However, if the incomplete genome of *L. zeae* DSM 20178 is included, its clusters together with *L. casei* ATCC 393 and next to the *rhamnosus* clade (Additional file 5).

*L. gallinarum* HFD4 clusters in the core-genome within the *helveticus* clade (Fig. 2 and Additional file 6). In the pan-genome, however, it clusters outside of the *helveticus* clade (Fig. 3). Analyses of the 16S rRNA gene sequence search of HDF4 revealed over 99% identity with the 16S rRNA gene sequence of various *L. helveticus* strains. *L. gallinarum* HFD4 contains 181 KOs that are not present in the 8 *L. helveticus* strains. Beside 135 hypotheticals, 10 KOs are associated with genetic information processing and 9 KOs with environmental information processing. Isolate HFD4 possesses an L-aspartate oxidase, an enzyme that converts L-aspartate to oxaloacetate and a DNA (cytoseine-5)-methyltransferase 1, which catalyzes the conversion from L-aspartate-4-semialdehyde to L-homoserine. However, these two KOs do not allow the strain to produce additional amino acids compared to the 8 *L. helveticus* strains. Additionally, isolate HFD4 contains macrolide transport system ATP-binding/permease protein (Table 3).

### Analysis of core- and pan-genome of the genus *Lactobacillus*

The metabolic capacity of the core- and pan-genome of the genus *Lactobacillus* was analyzed by using Brite protein family enrichment and pathway reconstruction in GhostKOALA. Reconstruction of protein families revealed an average increase of 6.1 fold from core- to pan-genome. A significant lower increase of 2.8-fold was observed in the class “genetic information processing” from core- to pan-genome and a significant higher increase of 17.9-fold in the “signaling and cellular processes” class (Table 4). The pathway reconstruction analysis revealed a 7.1-fold increase core- to pan-genome, a significant lower increase of 2.4-fold of genes in “genetic information processing” and a significant higher 24.9-fold increase for “Environmental information processing”, paralleling the observation in the protein family enrichment analysis.

**Table 4:** Reconstruction of core-, softcore- and pan-genome of the genus *Lactobacillus* with Brite and Pathway algorithm of GhostKOALA

Brite Reconstruction Result				n-fold increase	
	Core	Softcore	Pan	Core-pan	Softcore-pan
Orthologs and modules	237	471	1650	7.0	3.5^*^
Protein families: metabolism	180	320	1093	6.1_	3.4_
Protein families: genetic information processing	165	292	458	2.8^*^	1.6^*^
Protein families: signaling and cellular processes	27	73	484	17.9*	6.6^*^
Total	609	1156	3685	6.1_	3.2_
Pathway Reconstruction Result				n-fold increase	
	core	softcore	pan	core to pan	softcore-pan
Metabolism	303	502	2298	7.6^*^	4.6^*^
Genetic Information Processing	84	156	199	2.4^*^	1.3^*^
Environmental Information Processing	10	28	249	24.9^*^	8.9^*^
Cellular Processes	11	20	135	12.3_	6.8_
Organismal Systems	8	9	83	10.4_	9.2_
Human Diseases	18	33	109	6.1_	3.3_
Total	434	748	3073	7.1_	4.1_

^*^ indicates *p*-value < 0.01

### Core- and pan-genome of the type species *Lactobacillus delbrueckii*

**Fig. 4:**
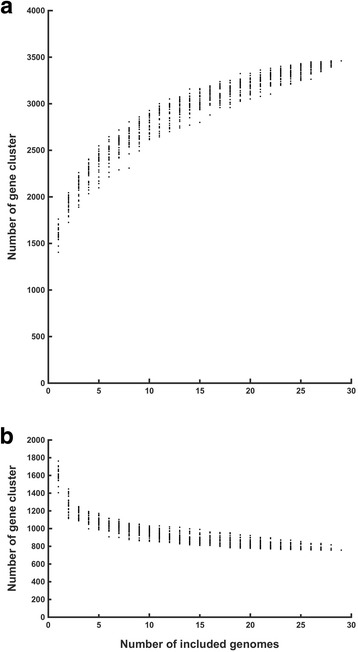
Pan- and core-genome evolution of *Lactobacillus delbrueckii*. **a** Evolution of the pan-genome for *L. delbrueckii*. After 20 included genomes, the pan-genome is closed. **b** Evolution of the core-genome for *L. delbrueckii*. Order of calculation was randomized for 29 sets, each represented with a single point

To gain insight in the core- and pan-genome of a *Lactobacillus* species, similar analyses as for the genus were performed with the type species of the genus: *Lactobacillus delbrueckii* (Additional files 7 and 8)*.* The *L. delbrueckii* core-genome contained 756 genes, the softcore-genome 1042 genes and the pan-genome 3460 genes. The average genome size was 1873 ± 93 genes (Table 1). The pan-genome of *L. delbrueckii* is gaining only 4–5 genes per genome after 26 included genomes and can be considered as closed (Fig. 4).

If *L. delbrueckii* MN-BM-F01, formerly *L. acidophilus* MN-BM-F01 [37], was excluded from the analyses, the core-genome increased only by 4 genes. This supports strongly the new classification of MN-BM-F01 from *L. acidophilus* to *L. delbrueckii*.

The quality criterion for genomes was set that the gene number should be within a range of ± 2σ around the average gene and protein number. If genomes that do not match this criterion were included in the analyses, e.g. the genomes of *L. delbrueckii* JCM1002, *L. delbrueckii* JCM1012 and *L. delbrueckii* CRL871, the core-genome dropped dramatically from 756 to 302 core genes, showing clearly the sensibility of the core genome for low quality sequenced genomes.

**Fig. 5:**
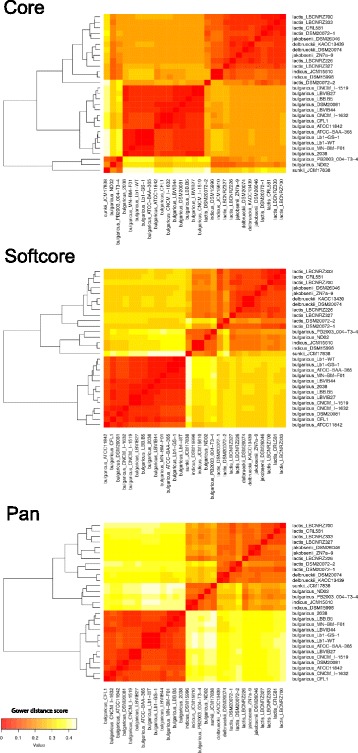
Heatmap of core-, softcore- and pan-genome for *L. delbrueckii.* In the core-genome heatmap the *bulgaricus* clade is embedded within the diverse clade. In the softcore- and pan-genome heatmap is the *bulgaricus* and the diverse clade are separated. Gower distance score based on ANI: Red = more similar, white = less similar

The 29 *L. delbrueckii* strains are separated in 2 clades in both the softcore- and pan-genome tree (Fig. 5). In the core-genome a small third clade containing the 3 strains PB2003_004-T3–4, ND02 and JCM17838 occurs. One clade in the pan-genome tree contains 13 strains that all belong to *Lactobacillus delbrueckii* subsp. *bulgaricus* and was therefore designated “bulgaricus” clade. The second clade, contains 16 isolates of the subspecies *Lactobacillus delbrueckii* subsp. *delbrueckii*, −*lactis*, −*indicus*, −*sunkii, −jakobsenii* and -*bulgaricus*, was designated “diverse” clade.

The average ANI over all *L. delbrueckii* genomes was 96.58 ± 0.93%. The average ANI in the bulgaricus clade was 98.05 ± 0.23% and in the diverse clade 96.23 ± 0.93%.

Core-genomes of both clades were constructed and the core genes were categorized with GhostKOALA. The bulgaricus clade core-genome contains 42 KOs that are not found in the diverse core-genome, of which 30 are hypothetical KOs. The 12 functionally annotated KOs are associated with carbon metabolism and environmental information processing (Table 2), including a complete sucrose-specific type II PTS system. There were, however, no functional differences between the two clades. This shows that evolutionary distinct genes with predicted identical functions are conserved in the strains in the clades. This is illustrated by the different aspartate kinases found in the 2 core genomes. Asparte kinase connects the glycine, serine and threonine metabolism with a number of other amino acid synthesis pathways. The enzymes in the bulgaricus clades are more than 79% identical to each other and the enzymes the diverse clades more than 95%. The two types of enzymes; however, have only an identity percentage of 34% or less and thus seem evolutionary distinct. This suggests that the *L. delbrueckii* subsp. *bulgaricus* is evolving differently in aspartate metabolism compared to the non-bulgaricus strains.

The diverse clade core-genome contains 9 KOs that are not present in the *bulgaricus* clade. Of these KOs, 5 encoded for hypothetical KOs, 3 for amino acid metabolism KOs and one small subunit ribosomal protein S21 (Table 3). Further, an α-glucoside transport system is uniquely present in the diverse cluster. This ABC transporter transports, amongst others, maltose.

### Analysis of core- and pan-genome of *Lactobacillus delbrueckii*

The metabolic capacity of the core- and pan-genome of *L. delbrueckii* was analyzed using protein family enrichment and pathway reconstruction in GhostKOALA. An increase of 1.8-fold from core- to pan-genome was measured with a significant lower increase of 1.4-fold in the class “genetic information processing” from core- to pan-genome and a significant higher 2.6 -fold increase in the “signaling and cellular processes” class (Additional file 9). In the pathway reconstruction, a significant lower increase of genes in “genetic information processing” and a higher increase for “Environmental information processing” was measured. These findings parallel the previous analysis the core- and pan-genome of the genus *Lactobacillus* (Table 4).

The reconstruction according to manually defined functional units, KEGG modules, revealed that short pathways are completely present in the core-genome whereas 1 or 2 enzymes were missing in many longer pathways (Additional file 10). However, many of such longer pathways such as the glycolysis, purine ribonucleotide biosynthesis, RNA polymerase, aminoacyl-tRNA biosynthesis and the ribosome protein complex are complete in the softcore. Taken together, fundamental processes in the cell are conserved in the softcore-genome and processes involved in interactions with the environment are only complete in the pan-genome.

### Analyses of core- genes of *L. delbrueckii*

To determine whether EcoSNPs in the core genes are responsible for the occurrence of the two clades in the core-genome tree, the consensus sequence for all 756 genes in the *L. delbrueckii* core-genome was calculated and SNPs in all 29 strains analyzed. In total, 53′583 SNPs were detected in all core genes. However, no cluster specific SNPs were detected, showing that the formation of 2 clades in the clustering is not dependent on a small set of EcoSNPs.

To analyze if all genes in the core genome had a similar phylogenetic tree, the tree of every gene in the core-genome was compared to the tree based on the core-genome (Fig. 5). The top 5% (*n* = 38) of genes with trees most similar to the core-genome tree had an average nodal distance score of 2.10 ± 0.13 and an average gene size of 1424 bp. The consensus sequences of the genes had an SNP density of 75.3 SNPs/kb. Of these 38 genes, 9 genes were interacting with DNA or RNA and there were no hypothetical genes (Additional file 11). The genes with trees least similar to the core-genome tree had an average nodal distance score of 6.47 ± 0.78, an average gene size of 407 bp. Of the 38 genes, 16 are either annotated as 30S or 50S ribosomal proteins. The consensus sequences of the 38 genes had an SNP density of 26.54 SNPs/kb, a density that is clearly lower than the average SNP density of 70.25 SNPs/kb. The 38 genes are thus highly homologous. This shows that highly conserved genes have a different phylogenetic tree than moderate conserved genes and such genes are not useful for phylogenetic reconstruction at species level.

### Potential HGT in *Lactobacillus delbrueckii*

To detect whether gene transfer appeared between the two clades in *L. delbrueckii*, we screened for potential HGT-genes within the two clades. In the *L. delbrueckii* pan-genome, a total of 57 genes were detected that were present in a subset of strains in both clades and are therefore potentially involved in HGT. 42 of those 57 genes encode for hypothetical proteins or are associated with phages or transposons (Additional file 12). Phages and transposons are commonly associated with HGT and their occurrence shows that our simple algorithm can detect HGT related genes.

### Core- and pan-genome of other *Lactobacillus* species

To determine if the type species *L. delbrueckii* is representative for other *Lactobacillus* species, we calculated the core- and pan-genome for four other species, one from each of the four clades observed in the core-genome clustering; *L. helveticus*, *L. rhamnosus*, *L. reuteri* and *L. plantarum* (Fig. 1). *L. helveticus* has a core-genome of 908 and pan-genome of 3350 genes with an average genome size of 2050 ± 164 genes (Table 1, Additional files 13 and 14). A similar ratio of core-genome to average genome size was calculated for *L. reuteri* with 897 core genes and 3960 pan genes on an average genome size of 2050 ± 117 genes (Table 1, Additional files 15 and 16). A lower ratio of core-genome to average genome size was calculated for *L. rhamnosus* with 811 core genes and 4889 pan genes on an average genome size of 2788 ± 71 genes (Table 1, Additional files 17 and 18). The core- and pan-genome for those three species are all closed (Additional files 19, 20 and 21). The biggest core-genome was calculated for the species *L. plantarum* with 1037 core-genes which is around 34% of the average genes in a *L. plantarum* genome (Table 1, Additional files 22 and 23). Neither the core- nor the pan-genome of *L. plantarum* were closed even after 122 genomes were included (Additional file 24).

### Clustering of core- and pan-genome of other *Lactobacillus* species

*L. rhamnosus* and *L. plantarum* clustered in two clades (Additional files 25 and 26), *L. reuteri* and *L. helveticus* in a number of minor clades (Additional files 27 and 28). The smaller *L plantarum* clade contained the type strain *L. plantarum* subsp. *argentoratensis* DSM 16365 and was designated the *argentoratensis* clade whereas the bigger clade contained the type strain *L. plantarum* subsp. *plantarum* ATCC 14917 and was designated the *plantarum* clade.

### Potential HGT in other *Lactobacillus* species

The species *L. plantarum* and *L. rhamnosus* cluster in 2 clearly separated clades and were used for HGT analyses. *L. plantarum* and *L. rhamnosus* possess 95 and 38 potential HGT genes in their pan-genome, respectively (Table 5). The majority of those genes encode hypothetical proteins. In *L. helveticus* only one gene was detected, a transposase, and in *L. reuteri* none*.*

**Table 5:** Gene annotation for potential HGT genes in *Lactobacillus* species

			Genes related to			
Organism	Clades	transferred genes	phage	transposon	hypothetical	others
*L. delbrueckii*	2	57	0	5	26	26
*L. helveticus*	3	1	0	1	0	0
*L. plantarum*	2	95	5	2	44	44
*L. reuteri*	2	0	0	0	0	0
*L. rhamnosus*	2	38	6	5	11	16

### HGT between clades in the genus *Lactobacillus*

Since we detected in four out of five analyzed species potential genes related to HGT, also the pan-genome of *Lactobacillus* genus was analyzed for HGT. The 20′800 pan genes of the genus *Lactobacillus* contains 2 genes occuring in all 4 clades with a probability of 30–70% (Table 6). Gene 1 encodes a type I restriction-modification system subunit M (ID = YP_004888889 in *L. plantarum* WCFS1) with a length of 539 aa. Gene 2 encodes a putative cell division protein (ADY84228 in *L. delbrueckii* 2038) with a length of 659 aa. Therefore, HGT occurs even between *Lactobacillus* species.

**Table 6:**
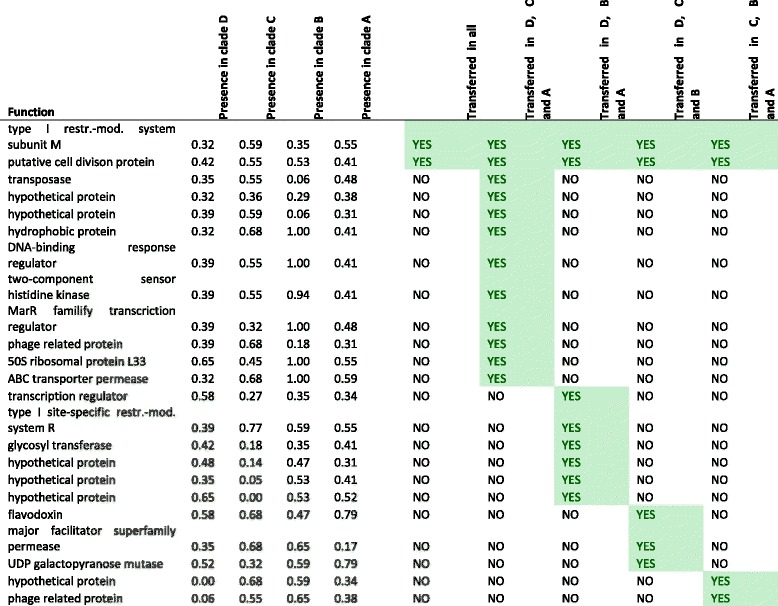
Gene annotation of potential HGT genes in the genus *Lactobacillus.* Genes that were potentially horizontally transferred within clades are marked in green

## Discussion

We clustered 98 complete sequenced genomes of 32 species of the genus *Lactobacillus* and calculated core- and pan-genome. The core-genome contained 266 genes. A core-genome of 175 *Lactobacillus* isolates and 26 strains from 8 *Lactobacillus*-related genera calculated with similar parameters presented a core-genome of only 73 genes [38]. The lower amount of core genes in the latter study is likely due to the higher number of genomes in the dataset, the integration of genomes from other genera, and including draft genomes in the analysis [18]. Especially incomplete or poorly assembled genomes have a large impact on the core-genome, as shown for core-genome of *L. delbrueckii* in this work. Since the core-genome is very sensitive to heterogeneous datasets and low sequence quality, a prior quality selection is necessary [16, 39]. The minimum standards for submitting a prokaryotic genome to Genbank are, amongst others, at least one copy of 5S, 16S and 23S rRNA-operon, a tRNA gene for each amino acid, and a ratio of genes to genome length close to 1 [40]. However, we showed that those standards are not restrictive enough for core-genome analysis and an additional selection of 2-fold the standard deviation of genes number was therefore used.

Another study using closed genomes revealed that the core-genome of 67 *Lactobacillus* strains from 25 species contained 311 genes [39]. The core-genome of *Lactobacillus* in our study was however, not closed after 67 genomes and between 290 and 406 genes (Fig. 1). The difference in the core-genomes is therefore likely due to the lower number of genomes in the previous study. The pan-genome of the *Lactobacillus* genus based on 67 strains contained 11′047 genes, clearly less than the pan-genome calculated in this study: 16148–18,318 genes for 67 genomes and 20′800 genes for 98 genomes. The larger pan-genome in our study is likely due to the more heterogenic dataset containing 32 species. Remarkably, the pan-genome of *Lactobacillus* is 4 times larger than the combined pan-genome of the narrow range genera *Staphylococcus* and *Macrococcus*. This exemplifies the wide habitat range and versatility of the *Lactobacillus* genus compared to *Staphylococcus* and *Macrococcus* [39, 41]. Moreover, the pan-genome of *Lactobacillus* was not closed after 98 genomes (Fig. 1). A closed pan-genome is rapidly reached in species that occur in a few habitats only or have a low capacity to acquire genes, such as *Bacillus anthracis* [42]*,* and, in this study, *L. delbrueckii.* Non-closed pan-genomes are typical for heterogeneous datasets, like the *Lactobacillus* dataset in this study, for species with diverse habitats, like *L. plantarum* in this study*,* and in species with high acquisition of genes, such as natural competent streptococci [16, 21]. Acquisition of genes occurs in lactobacilli occurs via HGT, which parallels observations in another genus frequently associated with the human gut: Bifidobacteria [43].

We analyzed the *Lactobacillus* type species *L. delbrueckii,* and the species *L. helveticus, L. reuteri, L. rhamnosus* and *L. plantarum* in more detail. The relative core-genome to average genome size was similar for all 5 species. In general, species with more genomes included in pan-genome analyses and higher genomic diversity, such as *L. plantarum,* have a smaller core-genome compared to the average genome size than species with less included genomes and a lower genomic diversity such as *L. delbrueckii* [44, 45]. A previous study revealed a core-genome of 2164 genes for 40 *L. rhamnosus* genomes [46] which is much higher than the 811 genes in our core-genome, yet close to soft-core genome of 1920 genes from 51 isolates (Table 1). Other studies revealed a *L. plantarum* core-genome of 1957 genes from 54 genomes and a *L. rhamnosus* core-genome of 2419 genes from 100 included genomes [47, 48], again much higher compared to the core-genomes in this study (Table 1). These core-genomes were; however, based on conserved function and not on sequence identity. The homologous-based comparison in this study is preferable, because it is based on true evolutionary events. This is clearly illustrated by the two clades in the core-genome of *L. delbrueckii*. The clades are clearly different from evolutionary view, but possess identical functional capacity (Table 3).

Analysis of the core- and pan-genome content revealed that fundamental processes like processing of genetic information and key metabolic pathways were conserved in the core-genome of *L. delbrueckii*, whereas environmental genes were not. These results are similar with compositions found in *S. aureus* [21] and *P. aeruginosa* [49], and parallels previous finding in lactobacilli [39].

In general, clustering of core- and pan-genome resulted in highly similar trees. Since the core genome contains the same genes for all isolates, the phylogenetic trees have to be based on information in the core-genome sequences. The strains of *Lactobacillus* clustered in species specific clusters (Fig. 1), with the exception of two strains: *L. casei* ATCC 393 and *L. gallinarum* HFD4. Differences of type strain *L. casei* ATCC 393 with other strains of *L. casei* are well documented [50–59]. The clustering in this study shows that ATCC 393 is most closely related to *L. zeae* DSM 20178 (Additional file 5), which confirms previous studies [53, 60]. However, a reclassification of type strain ATCC 393 as *L. zeae* was rejected by the Judicial Commission of the International Committee on Systematics of Bacteria [61]. Strain *L. gallinarum* HFD4 clustered different in core- and pan-genome clustering (Figs. 2 and 3). Genotypic differentiation for *L. gallinarum* and *L. helveticus* based on 16S rRNA sequence is not evident [62]. Initially, *L. gallinarum* and *L. helveticus* were differentiated based on their sugar fermentation pattern; *L. gallinarum* ferments amygdalin, cellobiose, salicin and sucrose, *L. helveticus* not [63]. However, none of the 181 KOs uniquely present in HDF4 encodes for any of these carbon sources and the phylogenetic differentiation between *L. helveticus* and *L. gallinarum* remains therefore unclear.

A separation in subspecies in the clustering of *L. delbrueckii* was already detected in a previous study based on MLST [64]. The separation is also visible in the ANI values within the clades, which were higher between members of the *bulgaricus* clade than between members of the mixed clade. Nevertheless, the ANI values were still above the cutoff value of 94% for different species [65] and all the analyses strains belong therefore to the same species.

Separation of populations into groups and further to species has been explained with several models. The infinitely many genes (IMG) model relates evolution and separation to all non-core-genome genes [66]. Since the *bulgaricus* clade separation already appears in the core-genome, the IMG model does not fit the evolution of *L. delbrueckii.* The ecotype model relates a mutation, identifiable as an ecoSNP, within a population to evolve into two subpopulations [67, 68]. EcoSNPs were not found in the *L. delbrueckii* analyses. However, ecoSNPs are only visible in recently diverged populations [12] whereas the division of *L. delbrueckii* into subspecies might not be recent. Convergent evolution was suggested in the genus *Lactobacillus* [69]. The example of two distinct aspartate kinases in the two clades of *L. delbrueckii* suggests convergent-like evolution. The aspartate kinase activity is; however, only an annotated function and it is possible that the two enzymes have different functions or activities in the cell, which would speak against convergent evolution.

The enrichment of environmental function in the accessory genome suggests that a *L. delbrueckii* population occupies a novel niche and then adapts via gene gain. In addition, gene exchange between the *L. delbrueckii* subpopulations occurred (Table 5). *L. delbrueckii* evolved therefore into subspecies with a mechanism that resembles the parapatric model used for specification in sexually reproducing organisms: a novel niche is occupied by a subpopulation that differentiates, but gene exchange with its original population is still possible.

The detection of HGT in *L. plantarum* and *L. rhamnosus* suggests they evolved similarly. Remarkably, *L. plantarum,* and *L. rhamnosus* were both considered as nomadic in a recent study [70] and such lifestyle provides opportunities for parapatric specification. *L. reuteri* and *L. helveticus* were not considered as nomadic [70] and indeed no evidence for parapatric differentiation in these species was found in our analyses.

## Conclusion

The sequenced based core- and pan-genome analyses of *Lactobacillus* and are useful to cluster and classify lactobacilli. The core- and pan-genome clustering yield similar trees. However, core-genomes clustering does not respect environmental adaptations, specific evolution or horizontal gene transfer. Pan-genome clustering was therefore necessary to show that *L. delbrueckii* evolved into subspecies via a parapatric-like model.

Our data provide novel insight how lactobacilli evolve and are related. This knowledge is useful for rational selection of strains for use in food fermentation.

## Additional files

Additional file 1: All strains used for core- and pan-genome analysis study. (XLSX 54 kb)

Additional file 2: Home-made scripts used in this study. (XLSX 39 kb)

Additional file 3: Core-genome fasta for genus *Lactobacillus* (Fasta). (XLSX 59 kb)

Additional file 4: Pan-genome fasta for genus *Lactobacillus* (Fasta). (XLSX 48 kb)

Additional file 5: Pan-genome clustering of genus *Lactobacillus.* Heatmap clustering according to 20,969 pan-genome genes from 99 *Lactobacillus* genomes including the non-complete genome of *L. zeae* DSM 20178. Gower distance score based on ANI: Red = more similar, white = less similar. *L. zeae* DSM 20178 marked with a red arrow. (FASTA 305 kb)

Additional file 6: Softcore-genome clustering of genus *Lactobacillus.* Heatmap clustering according to 594 softcore genes from 98 Lactobacillus genomes. Gower distance score based on ANI: Red = more similar, white = less similar. (FASTA 944 kb)

Additional file 7: Core-genome fasta for *Lactobacillus delbrueckii* (Fasta). (FASTA 278 kb)

Additional file 8: Pan-genome fasta for *Lactobacillus delbrueckii* (Fasta). (FASTA 1226 kb)

Additional file 9: Reconstruction of core, softcore- and pan-genome of *Lactobacillus delbrueckii* species with the Brite and pathway algorithm of GhostKOALA. (FASTA 219 kb)

Additional file 10: Reconstruction of core, softcore- and pan-genome of *Lactobacillus delbrueckii* species with the Module algorithm of GhostKOALA. Pathways are fractured in blocks (number). Green = pathway complete; yellow = 1 block is missing in the pathway; red = 2 or more blocks are missing in the pathway. (FASTA 1473 kb)

Additional file 11: TOPD/FMTS nodal distance scores and SNPs evaluation. Genes are sorted according to the nodal distance scores compared with the pan-genome tree and the 5% and 95% quantile is listed. Sum SNP – The sum of all SNP according to the consensus sequence of the 29 homologous sequences; SNP/base – sum of SNP divided by length of consensus sequence in nucleotide; Sum polyvariable positions (SPP) – sum of all positions with 3 or more different nucleotides a specific position; Length in bp – length of consensus sequence (JPEG 206 kb)

Additional file 12 Potential HGT in *Lactobacillus delbrueckii*. Clade B = *bulgaricus* clade, clade D = diverse clade, Number indicates copies of gene within the genome, presence = possibility of occurrence within the clade in percent. (TXT 2464 kb)

Additional file 13: Core-genome fasta for *Lactobacillus helveticus* (Fasta). (JPEG 215 kb)

Additional file 14: Pan-genome fasta for *Lactobacillus helveticus* (Fasta). (JPEG 289 kb)

Additional file 15: Core-genome fasta for *Lactobacillus reuteri* (Fasta). (FASTA 312 kb)

Additional file 16: Pan-genome fasta for *Lactobacillus reuteri* (Fasta). (FASTA 2283 kb)

Additional file 17: Core-genome fasta for *Lactobacillus rhamnosus* (Fasta). (JPEG 302 kb)

Additional file 18: Pan-genome fasta for *Lactobacillus rhamnosus* (Fasta). (PDF 3325 kb)

Additional file 19: Pan- and core-genome evolution of *L. helveticus.* **A** Evolution of the pan-genome for *L. helveticus*. After 14 included genomes, the pan-genome is closed. **B** Evolution of the core-genome for *L. helveticus*. Order of calculation was randomized for 19 sets, each represented with a single point. (PDF 5105 kb)

Additional file 20: Pan- and core-genome evolution of *L. reuteri.* **A** Evolution of the pan-genome for *L. reuteri*. After 20 included genomes, the pan-genome is closed. **B** Evolution of the core-genome for *L. reuteri*. Order of calculation was randomized for 25 sets, each represented with a single point. (PDF 2010 kb)

Additional file 21: Pan- and core-genome evolution of *L. rhamnosus.* **A** Evolution of the pan-genome for *L. rhamnosus*. After 20 included genomes, the pan-genome is closed. **B** Evolution of the core-genome for *L. rhamnosus*. Order of calculation was randomized for 51 sets, each represented with a single point. (PDF 1978 kb)

Additional file 22: Core-genome fasta for *Lactobacillus plantarum* (Fasta). (FASTA 193 kb)

Additional file 23: Pan-genome fasta for *Lactobacillus plantarum* (Fasta). (FASTA 5115 kb)

Additional file 24: Pan- and core-genome evolution of *L. plantarum.* **A** Evolution of the pan-genome for *L. plantarum*. The pan-genome remains open even after 122 genomes were included. **B** Evolution of the core-genome for *L. plantarum*. Order of calculation was randomized for 122 sets, each represented with a single point. (PDF 6945 kb)

Additional file 25: Pan-genome heatmap of *L. rhamnosus.* Heatmap clustering according to 4889 pan-genome genes from 51 *Lactobacillus rhamnosus* genomes. Gower distance score based on ANI: Red = more similar, white = less similar, strains with deviating cluster behavior marked with red arrows. (PDF 7940 kb)

Additional file 26: Pan-genome heatmap of *L. plantarum.* Heatmap clustering according to 7610 pan-genome genes from 122 *Lactobacillus plantarum* genomes. Gower distance score based on ANI: Red = more similar, white = less similar, strains with deviating cluster behavior marked with red arrows. (FASTA 469 kb)

Additional file 27: Pan-genome heatmap of *L. reuteri.* Heatmap clustering according to 3960 pan-genome genes from 25 *Lactobacillus reuteri* genomes. Gower distance score based on ANI: Red = more similar, white = less similar, strains with deviating cluster behavior marked with red arrows. (FASTA 1001 kb)

Additional file 28: Pan-genome heatmap of *L. helveticus.* Heatmap clustering according to 3350 pan-genome genes from 19 *Lactobacillus helveticus* genomes. Gower distance score based on ANI: Red = more similar, white = less similar, strains with deviating cluster behavior marked with red arrows. (XLSX 36 kb)

## Abbreviations

ANI: Average nucleotide identity
DDH: DNA-DNA hybridization
HGT: Horizontal gene transfer
KO: KEGG orthology
NGS: Next generation sequencing
OMCL: Ortho Markov Cluster algorithm
WGS: Whole genome sequencing
