# Supplementary figures and images for: Clustering of Pan- and Core-genome of Lactobacillus provides Novel Evolutionary Insights for Differentiation
Source: BMC Genomics. 2018 Apr 24;19:284. doi: 10.1186/s12864-018-4601-5 (PMC5937832; doi:10.1186/s12864-018-4601-5)

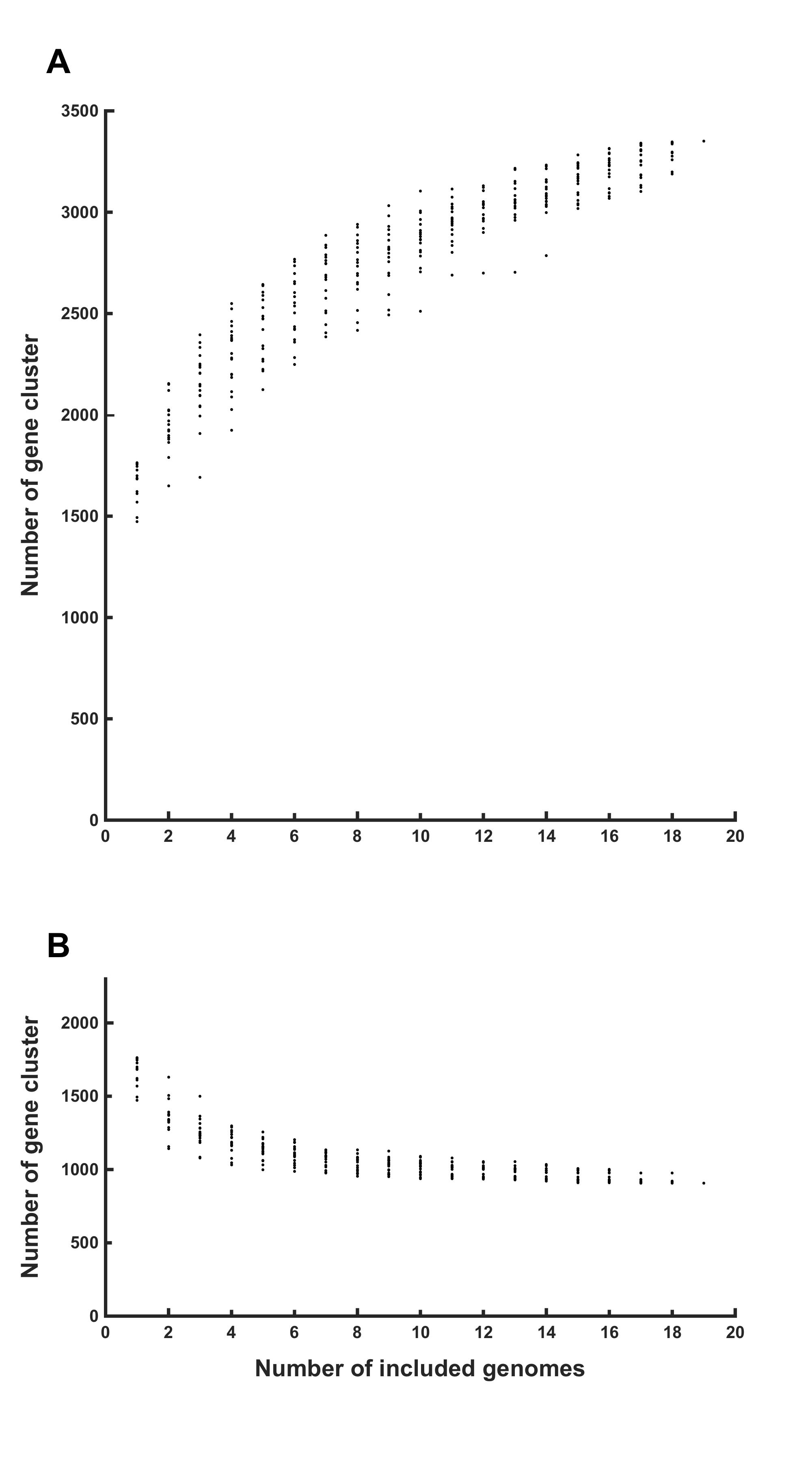

Supplement: Supplementary file 11 — TOPD/FMTS nodal distance scores and SNPs evaluation. Genes are sorted according to the nodal distance scores compared with the pan-genome tree and the 5% and 95% quantile is listed. Sum SNP – The sum of all SNP according to the consensus sequence of the 29 homologous sequences; SNP/base – sum of SNP divided by length of consensus sequence in nucleotide; Sum polyvariable positions (SPP) – sum of all positions with 3 or more different nucleotides a specific position; Length in bp – length of consensus sequence (JPEG 206 kb) [file 12864_2018_4601_MOESM11_ESM.jpg]

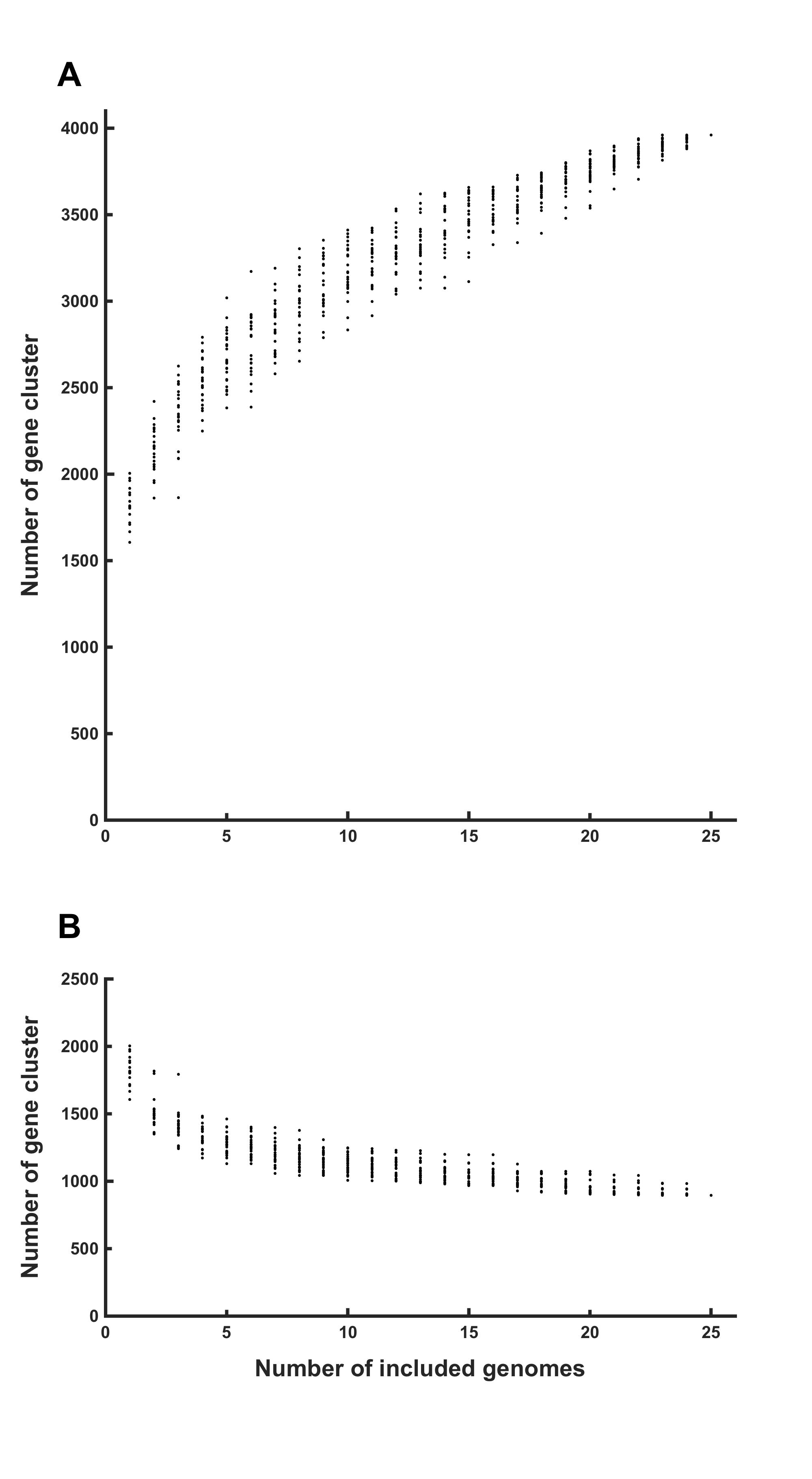

Supplement: Supplementary file 13 — Core-genome fasta for Lactobacillus helveticus (Fasta). (JPEG 215 kb) [file 12864_2018_4601_MOESM13_ESM.jpg]

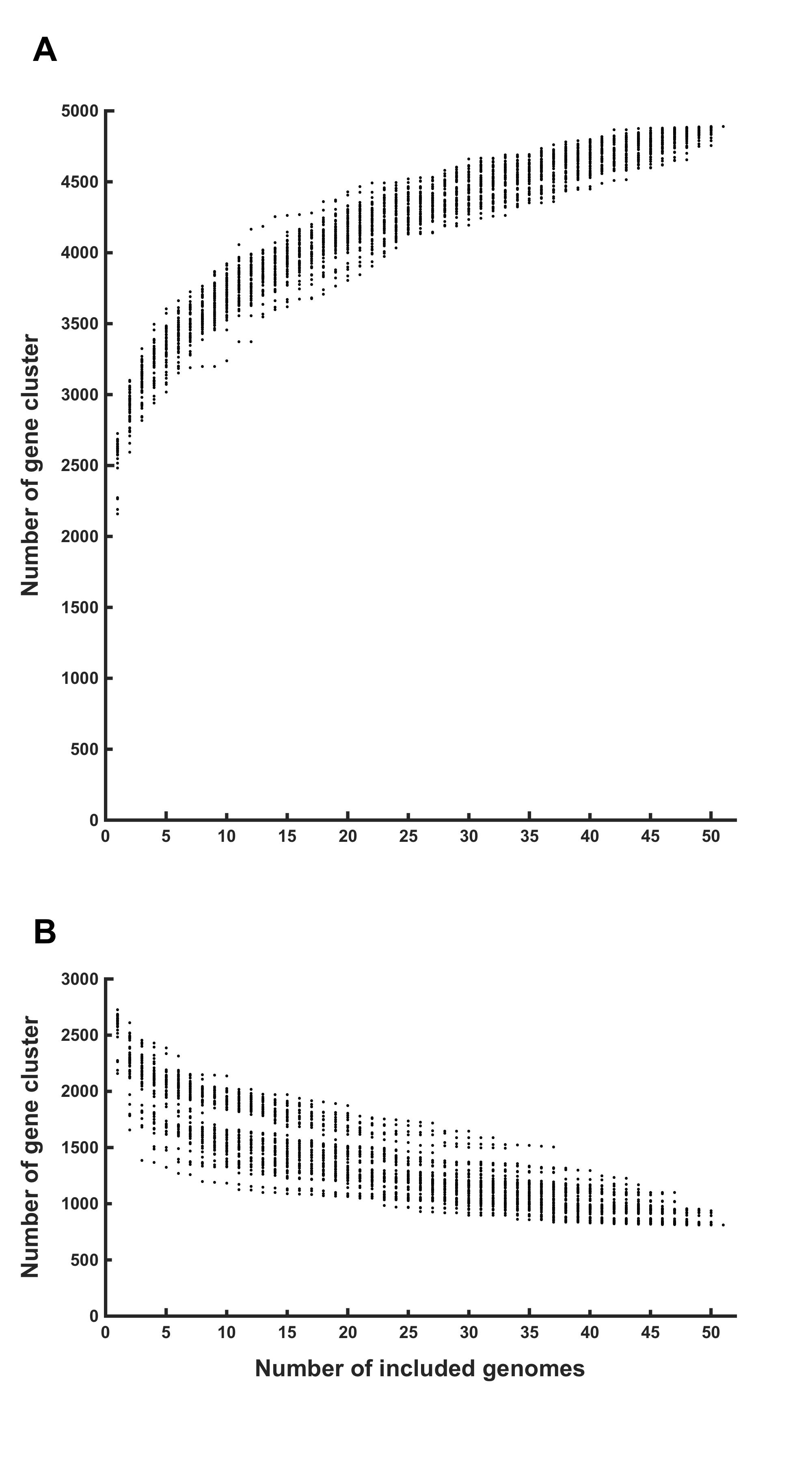

Supplement: Supplementary file 14 — Pan-genome fasta for Lactobacillus helveticus (Fasta). (JPEG 289 kb) [file 12864_2018_4601_MOESM14_ESM.jpg]

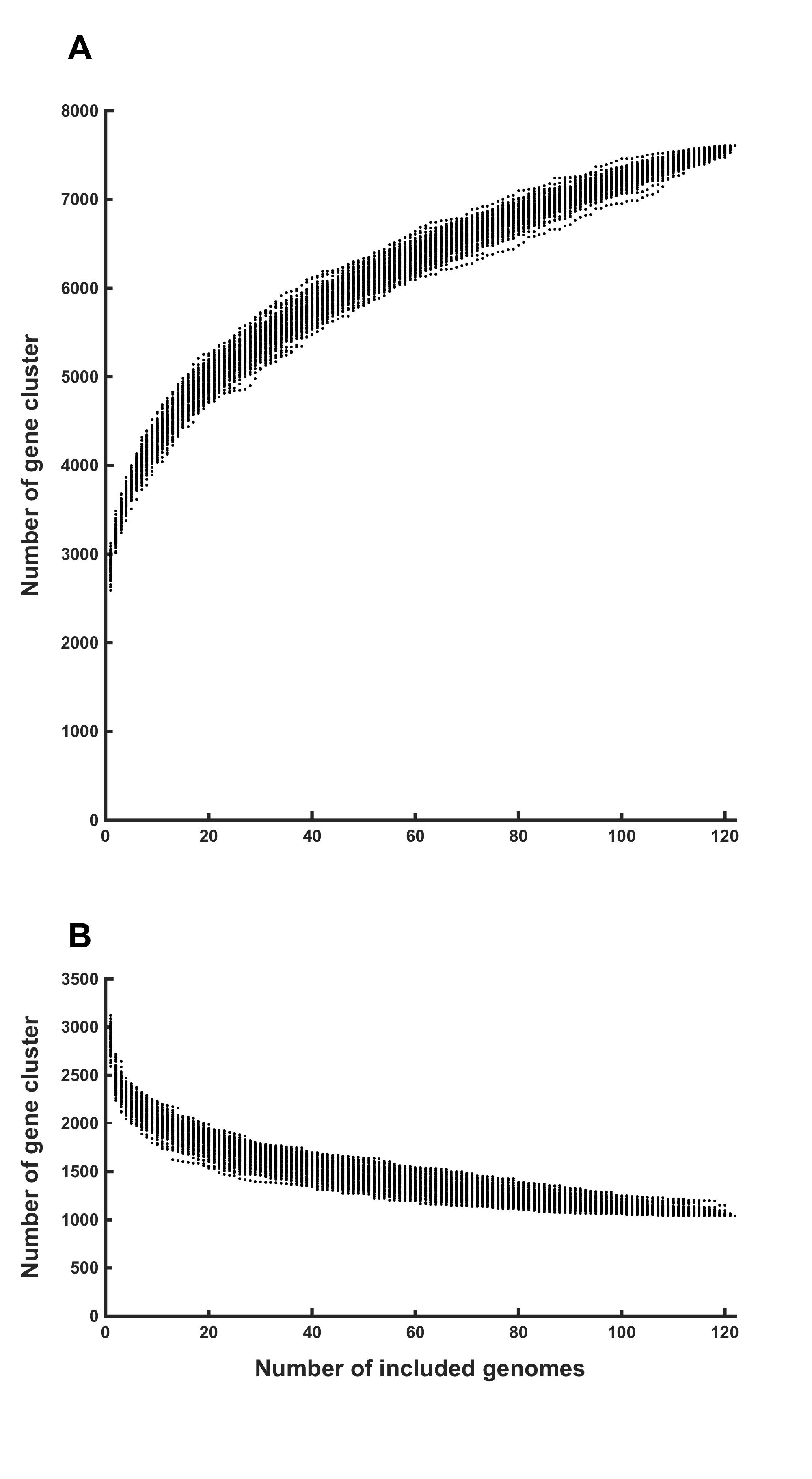

Supplement: Supplementary file 17 — Core-genome fasta for Lactobacillus rhamnosus (Fasta). (JPEG 302 kb) [file 12864_2018_4601_MOESM17_ESM.jpg]

gower distance score

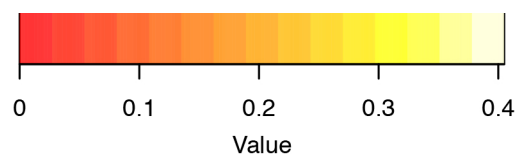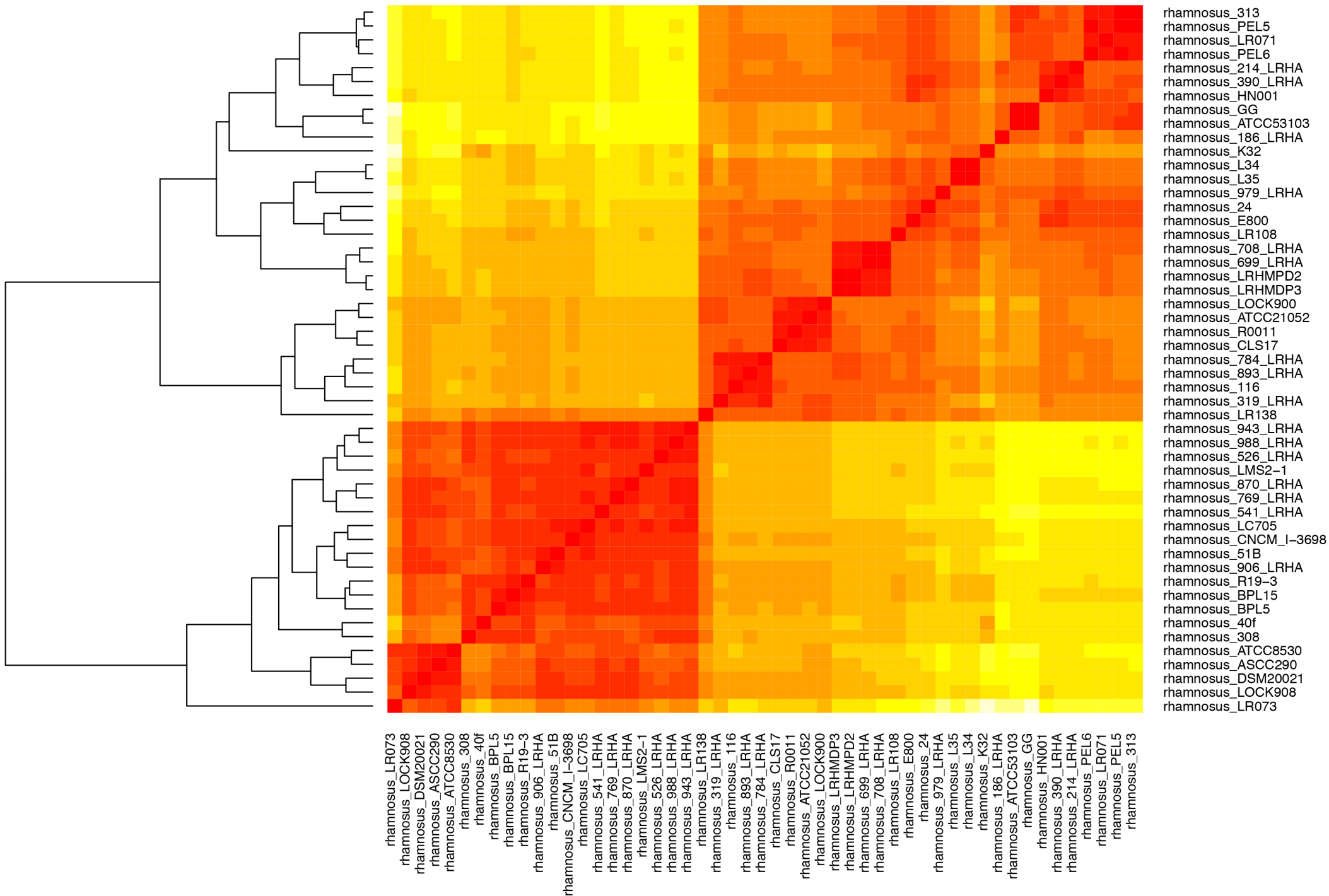

Supplement: Supplementary file 18 — Pan-genome fasta for Lactobacillus rhamnosus (Fasta). (PDF 3325 kb) [file 12864_2018_4601_MOESM18_ESM.pdf]

gower distance score

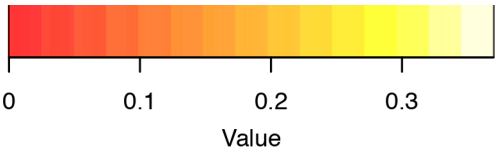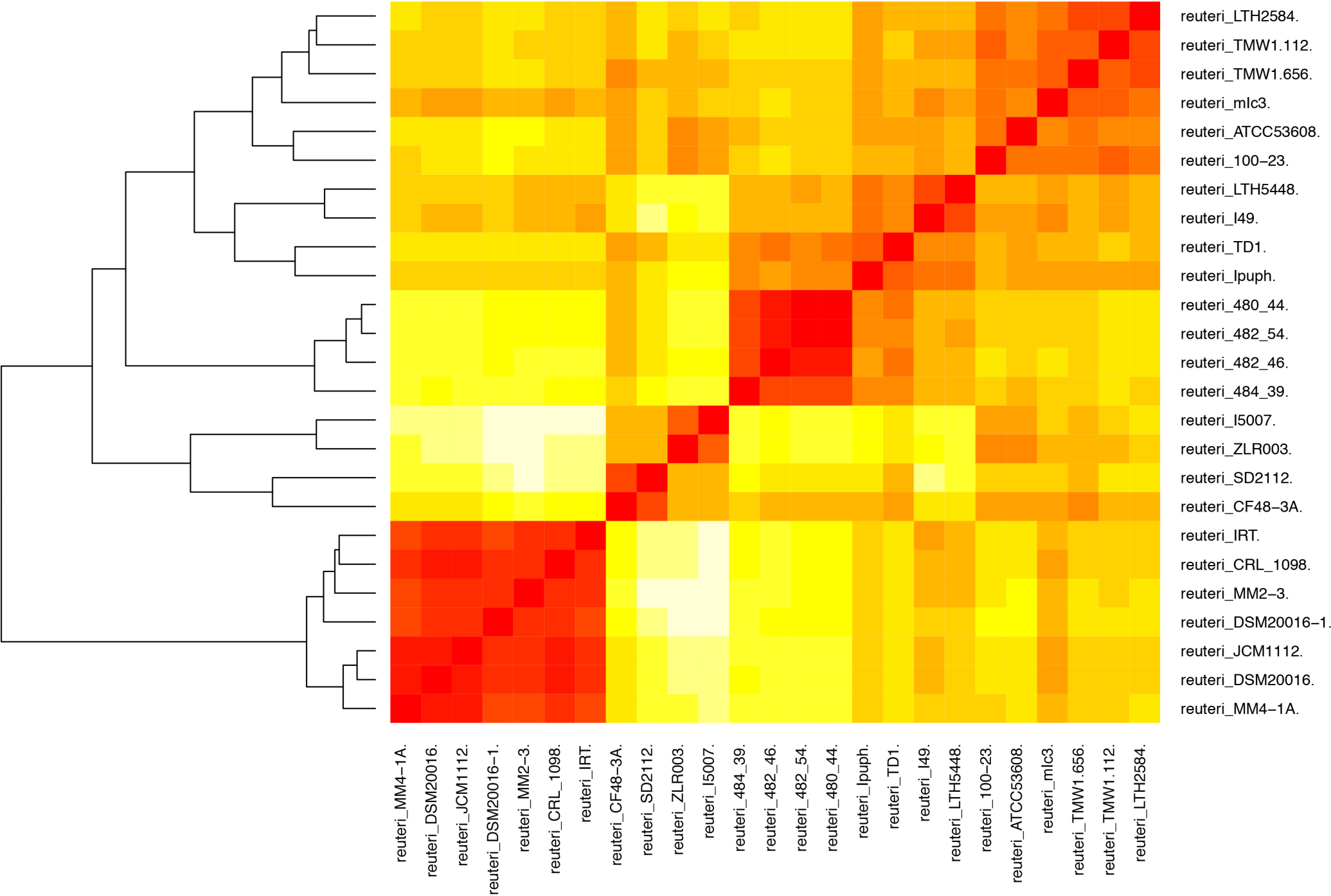

Supplement: Supplementary file 20 — Pan- and core-genome evolution of L. reuteri. A Evolution of the pan-genome for L. reuteri. After 20 included genomes, the pan-genome is closed. B Evolution of the core-genome for L. reuteri. Order of calculation was randomized for 25 sets, each represented with a single point. (PDF 2010 kb) [file 12864_2018_4601_MOESM20_ESM.pdf]

gower distance score

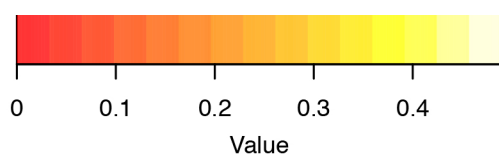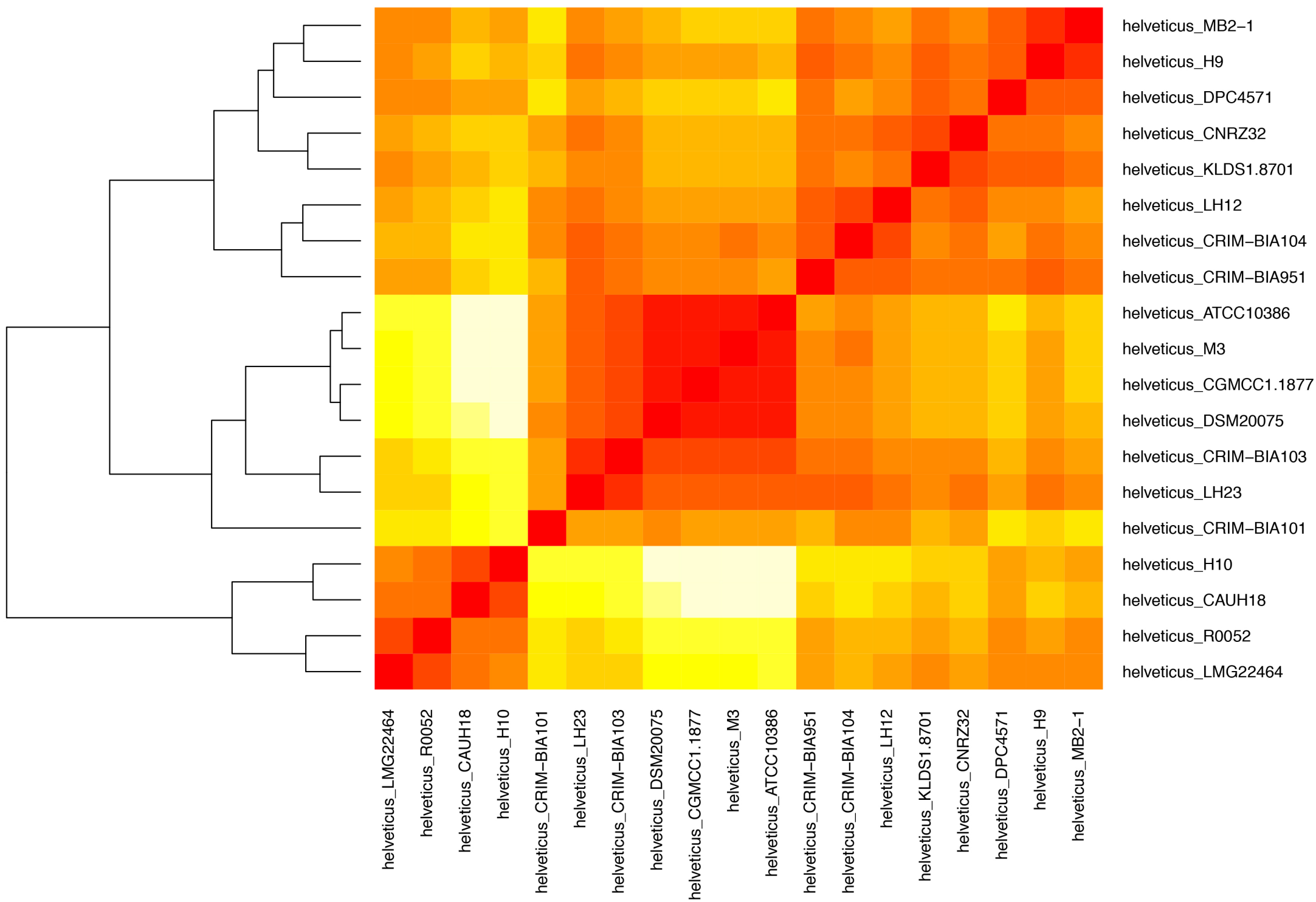

Supplement: Supplementary file 21 — Pan- and core-genome evolution of L. rhamnosus. A Evolution of the pan-genome for L. rhamnosus. After 20 included genomes, the pan-genome is closed. B Evolution of the core-genome for L. rhamnosus. Order of calculation was randomized for 51 sets, each represented with a single point. (PDF 1978 kb) [file 12864_2018_4601_MOESM21_ESM.pdf]

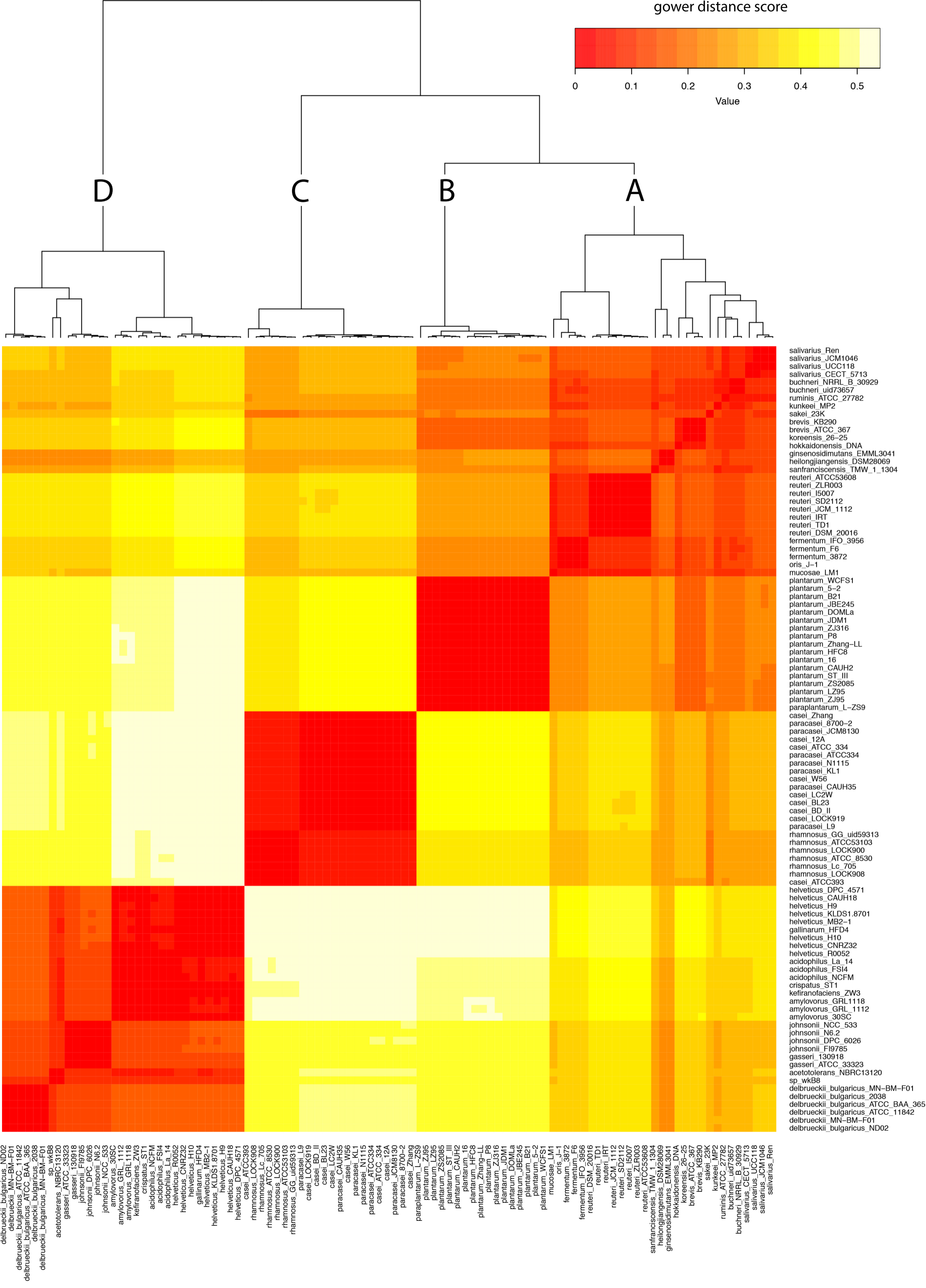

Supplement: Supplementary file 25 — Pan-genome heatmap of L. rhamnosus. Heatmap clustering according to 4889 pan-genome genes from 51 Lactobacillus rhamnosus genomes. Gower distance score based on ANI: Red = more similar, white = less similar, strains with deviating cluster behavior marked with red arrows. (PDF 7940 kb) [file 12864_2018_4601_MOESM25_ESM.pdf]
