# Supplementary material for: Clustering of Pan- and Core-genome of Lactobacillus provides Novel Evolutionary Insights for Differentiation
Source: BMC Genomics. 2018 Apr 24;19:284. doi: 10.1186/s12864-018-4601-5 (PMC5937832; doi:10.1186/s12864-018-4601-5)
Supplement: Supplementary file 19 — Pan- and core-genome evolution of L. helveticus. A Evolution of the pan-genome for L. helveticus. After 14 included genomes, the pan-genome is closed. B Evolution of the core-genome for L. helveticus. Order of calculation was randomized for 19 sets, each represented with a single point. (PDF 5105 kb) [file 12864_2018_4601_MOESM19_ESM.pdf]

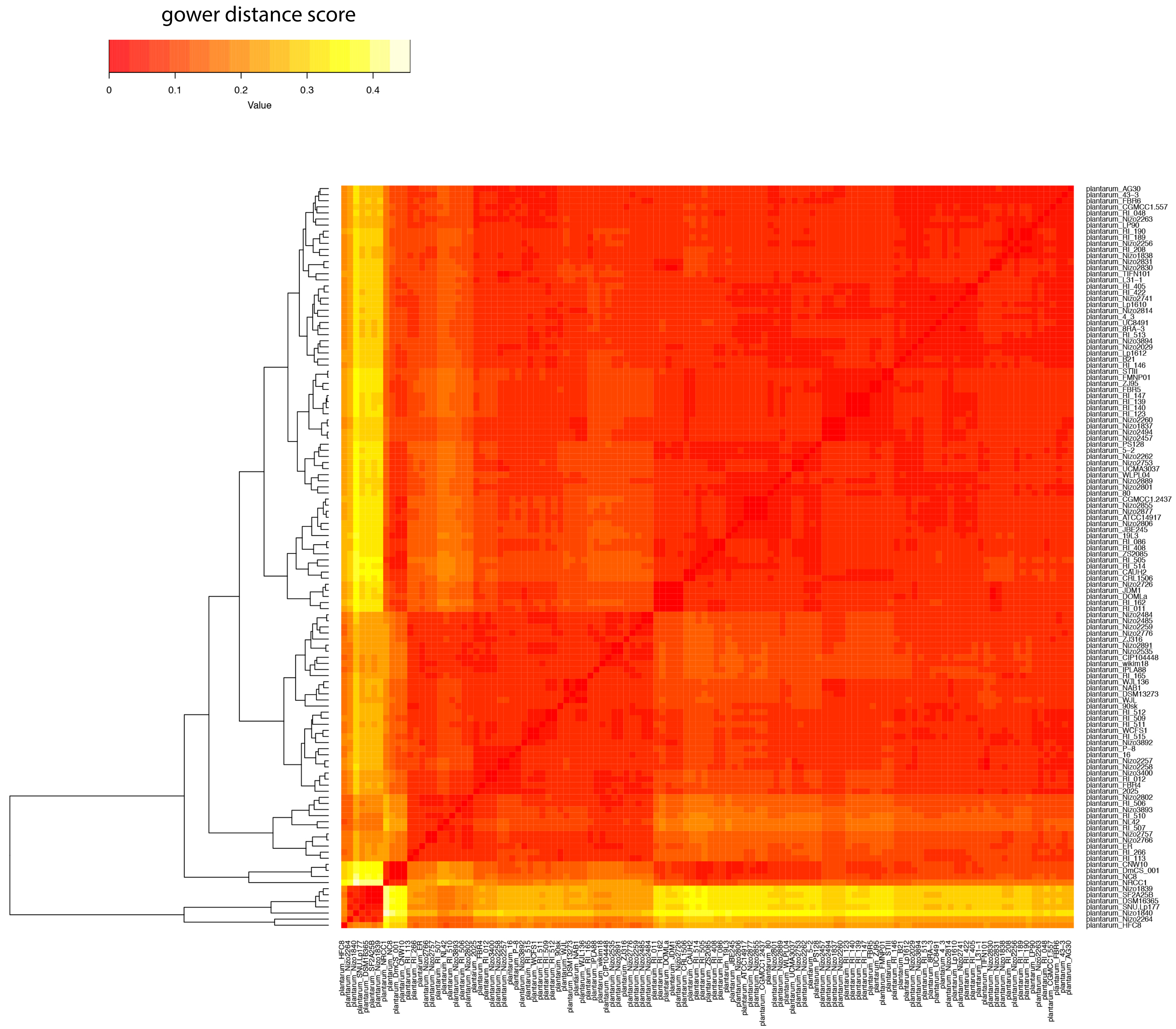
gower distance score
0
0.1
0.2
0.3
0.4
Value
plantarum_AG30
plantarum_43-3
plantarum_FBR6
plantarum_CGMCC1.557
plantarum_RI_048
plantarum_Nizo2263
plantarum_LP90
plantarum_RI_190
plantarum_RI_189
plantarum_Nizo2256
plantarum_RI_208
plantarum_Nizo1838
plantarum_Nizo2831
plantarum_Nizo2830
plantarum_TIFN101
plantarum_L31-1
plantarum_RI_405
plantarum_RI_422
plantarum_Nizo2741
plantarum_Lp1610
plantarum_Nizo2814
plantarum_4_3
plantarum_UC8491
plantarum_8RA-3
plantarum_RI_513
plantarum_Nizo3894
plantarum_Nizo2029
plantarum_Lp1612
plantarum_B21
plantarum_RI_146
plantarum_STIII
plantarum_FMNP01
plantarum_FBR5
plantarum_RI_147
plantarum_RI_139
plantarum_RI_140
plantarum_RI_123
plantarum_Nizo2260
plantarum_Nizo1837
plantarum_Nizo2494
plantarum_Nizo2457
plantarum_PS128
plantarum_5-2
plantarum_Nizo2262
plantarum_Nizo2753
plantarum_UCMA3037
plantarum_WLPL04
plantarum_Nizo2889
plantarum_Nizo2801
plantarum_80
plantarum_CGMCC1.2437
plantarum_Nizo2855
plantarum_Nizo2877
plantarum_ATCC14917
plantarum_Nizo2806
plantarum_JBE245
plantarum_19L3
plantarum_RI_086
plantarum_RI_408
plantarum_ZS2085
plantarum_RI_505
plantarum_RI_514
plantarum_CAUH2
plantarum_CRL1506
plantarum_Nizo2726
plantarum_JDM1
plantarum_DOMLa
plantarum_RI_162
plantarum_RI_011
plantarum_Nizo2484
plantarum_Nizo2485
plantarum_Nizo2259
plantarum_Nizo2776
plantarum_ZJ316
plantarum_Nizo2891
plantarum_Nizo2535
plantarum_CIP104448
plantarum_wikim18
plantarum_IPLA88
plantarum_RI_165
plantarum_WJL136
plantarum_NAB1
plantarum_DSM13273
plantarum_WJL
plantarum_90sk
plantarum_RI_512
plantarum_RI_509
plantarum_RI_511
plantarum_WCFS1
plantarum_RI_515
plantarum_Nizo3892
plantarum_P-8
plantarum_16
plantarum_Nizo2257
plantarum_Nizo2258
plantarum_Nizo3400
plantarum_RI_012
plantarum_FBR4
plantarum_2025
plantarum_Nizo2802
plantarum_RI_506
plantarum_Nizo3893
plantarum_RI_510
plantarum_NL42
plantarum_RI_507
plantarum_Nizo2757
plantarum_Nizo2766
plantarum_ER
plantarum_RI_266
plantarum_RI_113
plantarum_CNW10
plantarum_DmCS_001
plantarum_NC8
plantarum_NRCC1
plantarum_Nizo1839
plantarum_SF2A25B
plantarum_DSM16365
plantarum_SNU.Lp177
plantarum_Nizo1840
plantarum_Nizo2264
plantarum_HFC8
