# Supplementary material for: Clustering of Pan- and Core-genome of Lactobacillus provides Novel Evolutionary Insights for Differentiation
Source: BMC Genomics. 2018 Apr 24;19:284. doi: 10.1186/s12864-018-4601-5 (PMC5937832; doi:10.1186/s12864-018-4601-5)
Supplement: Supplementary file 24 — Pan- and core-genome evolution of L. plantarum. A Evolution of the pan-genome for L. plantarum. The pan-genome remains open even after 122 genomes were included. B Evolution of the core-genome for L. plantarum. Order of calculation was randomized for 122 sets, each represented with a single point. (PDF 6945 kb) [file 12864_2018_4601_MOESM24_ESM.pdf]

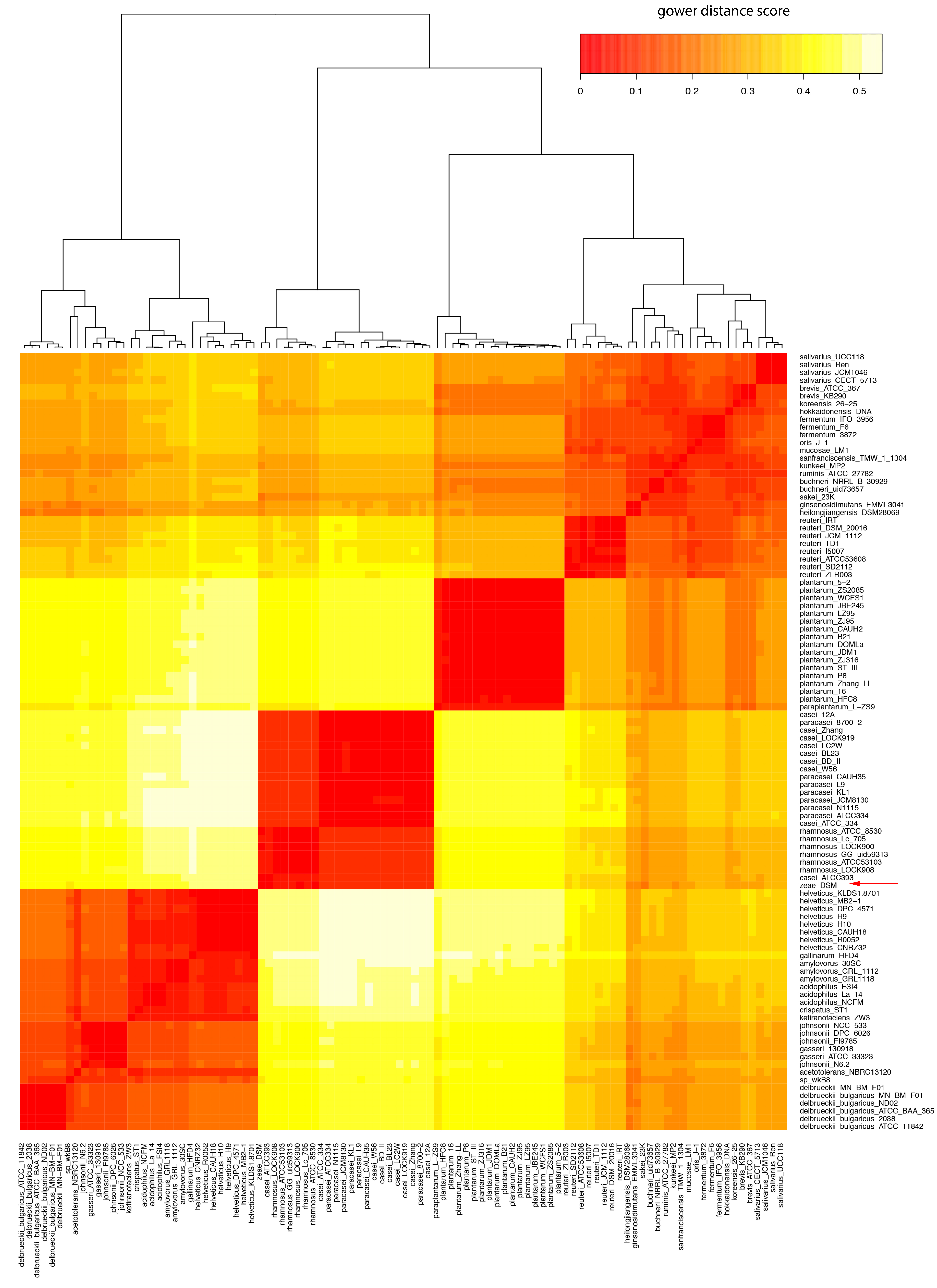
gower distance score
0
0.1
0.2
0.3
0.4
0.5
salivarius_UCC118
salivarius_Ren
salivarius_JCM1046
salivarius_CECT_5713
brevis_ATCC_367
brevis_KB290
koreensis_26–25
hokkaidonensis_DNA
fermentum_IFO_3956
fermentum_F6
fermentum_3872
oris_J–1
mucosae_LM1
sanfranciscensis_TMW_1_1304
kunkeei_MP2
ruminis_ATCC_27782
buchneri_NRRL_B_30929
buchneri_uid73657
sakei_23K
ginsenosidimutans_EMML3041
heilongjiangensis_DSM28069
reuteri_IRT
reuteri_DSM_20016
reuteri_JCM_1112
reuteri_TD1
reuteri_I5007
reuteri_ATCC53608
reuteri_SD2112
reuteri_ZLR003
plantarum_5–2
plantarum_ZS2085
plantarum_WCFS1
plantarum_JBE245
plantarum_LZ95
plantarum_ZJ95
plantarum_CAUH2
plantarum_B21
plantarum_DOMLa
plantarum_JDM1
plantarum_ZJ316
plantarum_ST_III
plantarum_P8
plantarum_Zhang–LL
plantarum_16
plantarum_HFC8
paraplantarum_L–ZS9
casei_12A
paracasei_8700–2
casei_Zhang
casei_LOCK919
casei_LC2W
casei_BL23
casei_BD_II
casei_W56
paracasei_CAUH35
paracasei_L9
paracasei_KL1
paracasei_JCM8130
paracasei_N1115
paracasei_ATCC334
casei_ATCC_334
rhamnosus_ATCC_8530
rhamnosus_Lc_705
rhamnosus_LOCK900
rhamnosus_GG_uid59313
rhamnosus_ATCC53103
rhamnosus_LOCK908
casei_ATCC393
zeae_DSM
helveticus_KLDS1.8701
helveticus_MB2–1
helveticus_DPC_4571
helveticus_H9
helveticus_H10
helveticus_CAUH18
helveticus_R0052
helveticus_CNRZ32
gallinarum_HFD4
amylovorus_30SC
amylovorus_GRL_1112
amylovorus_GRL1118
acidophilus_FSI4
acidophilus_La_14
acidophilus_NCFM
crispatus_ST1
kefiranofaciens_ZW3
johnsonii_NCC_533
johnsonii_DPC_6026
johnsonii_FI9785
gasseri_130918
gasseri_ATCC_33323
johnsonii_N6.2
acetotolerans_NBRC13120
sp_wkB8
delbrueckii_MN–BM–F01
delbrueckii_bulgaricus_MN–BM–F01
delbrueckii_bulgaricus_ND02
delbrueckii_bulgaricus_ATCC_BAA_365
delbrueckii_bulgaricus_2038
delbrueckii_bulgaricus_ATCC_11842
